# Generative design of compounds with desired potency from target protein sequences using a multimodal biochemical language model

**DOI:** 10.1186/s13321-024-00852-x

**Published:** 2024-05-22

**Authors:** Hengwei Chen, Jürgen Bajorath

**Affiliations:** Department of Life Science Informatics and Data Science, B-IT, Lamarr Institute for Machine Learning and Artificial Intelligence, LIMES Program Chemical Biology and Medicinal Chemistry, Rheinische Friedrich-Wilhelms-Universität, Friedrich-Hirzebruch-Allee 5/6, 53115 Bonn, Germany

**Keywords:** Deep learning, Molecular design, Protein language model, Conditional transformer, Active compounds

## Abstract

**Abstract:**

Deep learning models adapted from natural language processing offer new opportunities for the prediction of active compounds via machine translation of sequential molecular data representations. For example, chemical language models are often derived for compound string transformation. Moreover, given the principal versatility of language models for translating different types of textual representations, off-the-beaten-path design tasks might be explored. In this work, we have investigated generative design of active compounds with desired potency from target sequence embeddings, representing a rather provoking prediction task. Therefore, a dual-component conditional language model was designed for learning from multimodal data. It comprised a protein language model component for generating target sequence embeddings and a conditional transformer for predicting new active compounds with desired potency. To this end, the designated “biochemical” language model was trained to learn mappings of combined protein sequence and compound potency value embeddings to corresponding compounds, fine-tuned on individual activity classes not encountered during model derivation, and evaluated on compound test sets that were structurally distinct from training sets. The biochemical language model correctly reproduced known compounds with different potency for all activity classes, providing proof-of-concept for the approach. Furthermore, the conditional model consistently reproduced larger numbers of known compounds as well as more potent compounds than an unconditional model, revealing a substantial effect of potency conditioning. The biochemical language model also generated structurally diverse candidate compounds departing from both fine-tuning and test compounds. Overall, generative compound design based on potency value-conditioned target sequence embeddings yielded promising results, rendering the approach attractive for further exploration and practical applications.

**Scientific contribution:**

The approach introduced herein combines protein language model and chemical language model components, representing an advanced architecture, and is the first methodology for predicting compounds with desired potency from conditioned protein sequence data.

## Introduction

In drug discovery, compound optimization requires the comprehensive evaluation of multiple physicochemical and in vivo properties such as affinity, hydrophobicity, solubility, toxicity, pharmacogenetics, and pharmacodynamics [1]. Experimental efforts to assess and optimize these molecular properties are supported by computational approaches [2], with quantitative structure–activity relationship (QSAR) analysis being a classical methodology for compound affinity prediction [3, 4], mostly focusing on congeneric compounds and progression of hit-to-lead or lead series.

In recent years, machine learning (ML) including deep learning (DL) has increasingly been considered for activity and property predictions in drug discovery [5], leading to the application of various neural network (NN) methods such as convolutional NN (CNN) [6], recurrent neural NN (RNN) [7], graph convolutional network (GCN) [8], or message passing NN (MPNN) [9]. DL methods including those employed for property predictions generally benefit from the availability of large data sets for learning the multitude of internal weights they require. However, such data sets are for the most part unavailable in early-phase drug discovery where data sparseness often hinders the use of DL models and limits the accuracy of their predictions [10]. In addition, the assessment of ML methods for quantitative compound potency predictions in typical benchmark settings poses considerable challenges. Notably, benchmark potency predictions by ML/DL models of varying complexity and randomized predictions are often only differentiated by small error margins [11], thus complicating an unambiguous assessment of relative method performance [11]. As a consequence of data sparseness and intrinsic limitations in method evaluation and comparison, there currently are no generally applicable criteria or guidelines available for prioritizing ML approaches for quantitative molecular property predictions in drug discovery.

Property predictions can also be combined with generative modeling of new compounds [12], which provides a conceptual alternative to conventional property prediction strategies. For example, to this end, we have developed specialized transformer models, as further detailed below. In computer science, transformers originated from the field of natural language processing where they were used for the conversion of an input sequence of characters into an output sequence with the aid of self-attention (importance) mechanisms [13]. Transformer architectures are increasingly employed in other fields for various machine translation tasks. A transformer-based compound design concept investigated in our laboratory was semi-quantitative in nature. It aimed at deriving models for predicting potent compounds for targets of interest without specifying numerical potency values across wide ranges, thereby circumventing some of the obstacles associated with benchmark compound potency predictions [11]. Previously, we derived transformer-based chemical language models (CLMs) for molecular string-to-string conversion conditioned on potency differences between pairs of structural analogues [14, 15]. So-called conditional transformer models not only learn conditional probabilities for character sequence translation, but also for other context-dependent rules (such as molecular property constraints). Our rules included potency difference thresholds required for the formation of activity cliffs (i.e., analogue pairs having largest potency differences in compound activity classes) [14] or -in a generalized form- desired potency difference thresholds structural analogues [15]. In the latter case, transformer models were trained based on large numbers of analogue pairs with greatly varying potency differences. In both instances, conditional transformers consistently reproduced highly potent compounds from activity cliffs or other compound pairs for a variety of activity classes, thus providing proof-of-principle, and generated other structurally diverse candidate compounds [14, 15]. On the basis of these findings, we extended this transformer architecture for generative modeling of potent compounds by a meta-learning framework for modeling in low compound data regimes [16].

In addition to learning compound-to-compound mappings for predicting new active or highly potent compounds, various attempts have been made to establish direct links between biological targets and chemical entities with DL models using representations combining protein sequence and compound information [17–22]. These models were often derived to distinguish true target-ligand complexes from false (randomly assembled) complexes. Potential applications of such models include target validation or compound repurposing. Furthermore, in recent studies, transformer-based language models have been employed to learn mappings of protein sequences to compounds [22–25]. In the following, models using protein sequence data as input are termed protein language models (PLMs), regardless of the nature of the output sequences. Sequence-to-compound modeling aimed to revitalize the concept of sequence-based compound design [22] that was investigated during the early days of drug design but was then for long out of fashion in drug discovery settings, for scientific reasons. Notably, only limited numbers of residues in protein sequences are typically implicated in ligand binding and only high global sequence similarity indicates similar ligand binding characteristics of targets. Hence, designing active compounds based on sequence data is challenging and partly controversial, perhaps not even possible without additional knowledge, and difficult to pursue using standard ML methods. However, the advent of PLMs has made it possible to have a fresh look at this scientifically provoking design task. For example, a transformer was adapted to associate the primary structures of target proteins with known active compounds and predict new ones [23]. Compounds were represented as Simplified Molecular Input Line Entry System (SMILES) strings [26], a mainstay textual representation. In another study, an Lmser network-based transformer variant incorporating multi-head cross attention blocks was developed to map complete protein sequences to active compounds [24]. The encoder processed information from the protein sequence and the resulting latent space was decoded into compound SMILES. In addition, compound generation was combined with Monte Carlo tree search [24]. In both of these studies, conventional protein–ligand docking scores were used to guide compound prioritization. In a different investigation, a transformer was derived to associate extended sequence motifs of ligand binding sites with active compounds [25]. In this case, the ability of the model to exactly reproduce ATP site-directed inhibitors of different kinases not included in model training was used as a proof-of-concept criterion (instead of hypothetical scoring). Notably, the definition of sequence motifs directly implicated in compound binding requires prior (structural) knowledge.

Following principles from natural language processing, PLMs embed long protein sequences as sentences of characters in which one or more residues form words [27, 28]. The resulting sequence embeddings are thought to implicitly capture much information concerning structural and functional characteristics of proteins, rendering these embeddings attractive for a variety of applications [29, 30].

Given our previous studies of chemical language models for predicting potent compounds and the applications of PLMs discussed above, we have been interested in exploring the possibility to combining these approaches and investigating whether compounds with pre-defined potency could also be designed using a conditional transformer architecture and protein sequence data. To this end, we have developed and assessed a new so-called biochemical language model for learning from multimodal data, as presented in the following.

## Methods

### Targets, compounds, and activity data

Compounds with high-confidence activity data were selected from ChEMBL (release 33) [31]. Only compounds engaged in direct interactions (assay relationship type: "D") with human targets at the highest assay confidence level (assay confidence score 9) were considered. Potency measurements were restricted to numerically specified equilibrium constants (K_i_ values) and recorded as negative logarithmic pK_i_ values. In cases where multiple measurements were available for the same compound, the geometric mean was calculated as the final potency annotation, contingent on all values falling within the same order of magnitude; otherwise, the compound was excluded from further consideration. Qualifying compounds were divided into target-based activity classes. Only targets with a maximal (monomer) sequence length of 4000 residues were considered. On the basis of these data curation criteria, 1575 activity classes were obtained, comprising a total of 87,839 unique compounds. For each activity class, the protein sequence of the target was extracted in FASTA format from UniProt [32] using an in-house script. Compounds were represented as canonical SMILES strings generated using RDKit [33]. From the large activity class pool, 10 classes with at least close to 400 compounds were randomly selected as test cases for generative design (Table 1). These activity classes included ligands G protein-coupled receptors and inhibitors of different enzymes.

**Table 1:** Activity classes for model evaluation

ChEMBL ID	Target name	Compounds
204	Thrombin	454
218	Cannabinoid CB1 receptor	1118
234	Dopamine D3 receptor	1529
244	Coagulation factor X	702
251	Adenosine A2a receptor	1825
1862	Tyrosine-protein kinase ABL	499
4005	PI3-kinase p110-alpha subunit	576
5113	Orexin receptor 1	1086
1,075,104	Leucine-rich repeat serine/threonine-protein kinase 2	397
1,908,389	Mitogen-activated protein kinase kinase kinase 12	404

For each of 10 activity classes, the number of compounds, ChEMBL target ID, and target name are reported

### Model architecture

**Fig. 1:**
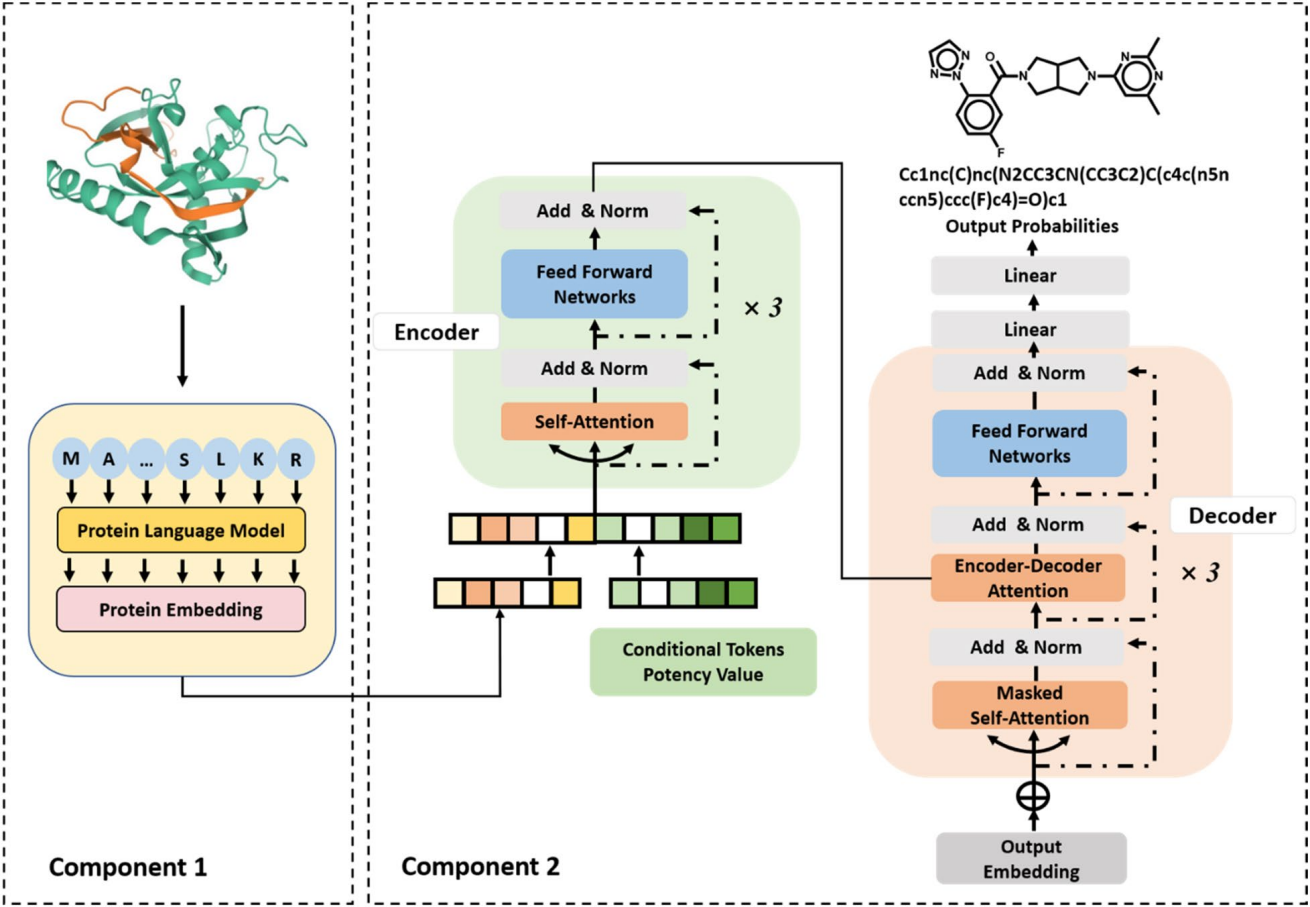
Architecture of the biochemical language model

For our prediction task, we devised a new multimodal conditional compound generator combining two language model components. Its characteristic feature is the design of compounds with desired potency based on protein sequence information conditioned on compound potency values. To our knowledge, this scheme represents a previously unconsidered design concept and, in addition, the first instance of a language model conditioned on molecular context rules from chemistry applied to biological sequences (thus also incorporating multimodality). The model architecture is schematically depicted in Fig. 1. A pre-trained PLM generating protein sequence embeddings (component 1) was combined with a conditional transformer (component 2) challenged to learn mappings of combined protein and potency values embeddings to compounds (SMILES strings) with corresponding activity against a given target. Accordingly, the transformer should predict compounds from target sequence embeddings having a desired potency level. Since the generator bridges between protein sequence information with compound activity constraints and chemical structure, it is termed a “multimodal biochemical language model”. In the following, the two model components are described in more detail.

### Protein language model for generating embeddings

Sequence embeddings should capture distributions of vast numbers of amino acid sequences of proteins, residue frequencies, and positional dependencies. Hence, they should implicitly encode characteristic features related to biophysical properties, structure, and function. For our study, we adapted as model component 1 the pre-trained ProtT5XLUniref50 PLM from ProtTrans [29] with default dimensionality of 1024. ProtTrans PLMs were originally derived based on ultra-large sequence data sets from UniRef [34] and BFD [35], comprising up to 2122 million proteins and 393 billion amino acids. Each protein sequence was initially tokenized and then subjected to positional encoding. The resulting vector was processed to generate context-aware embeddings for each input token (amino acid). These embeddings, extracted from the last hidden state of a PLM's attention stack, were concatenated and pooled along the length dimension. This pooling approach generated a fixed-size embedding, regardless of the input length [29]. ProtTrans embeddings are considered one of the pioneering developments in the field. In our work, ProtT5XLUniref50 protein embeddings of constant dimensionality were generated for each target and concatenated with conditional token embeddings representing compound potency values (see below). The resulting combined embedding vectors provided the input for the encoder of the conditional transformer (model component 2). The ProtTrans PLM was only used for calculating protein sequence embeddings and not involved in model derivation, optimization, or fine-tuning.

### Conditional transformer

The architecture of the conditional transformer was adapted from our previous study predicting highly potent compounds from weakly potent templates [15] and modified for generative design of compounds based on sequence data. The transformer was implemented using PyTorch [36]. It consisted of three encoder and three decoder modules with self-attention mechanism. Each encoder module included a multi-head self-attention sub-layer and a fully connected feed-forward neural network sub-layer. The encoder converted the input embedding into a context vector in its final hidden state, serving as input for the decoder. Each decoder contained two multi-head self-attention sub-layers and a feed-forward sub-layer. It transformed the context vector into a sequence of tokens. The masked self-attention sublayer processed the output of the preceding attention sub-layer to prevent translation errors. Compounds were predicted from a given protein sequence embedding conditioned on desired potency via the following triple:

(*Protein sequence embedding, Potency embedding*) → (*Compound*).

For a given protein sequence, representation vectors of the sequence embedding were initially computed using the ProtTrans PLM. Subsequently, the output protein embedding was concatenated with the potency embedding, forming combined representations as input for transformer encoder that were converted into a latent representation. The decoder then iteratively generated an output SMILES sequence until the stop token was obtained. Multinomial sampling was employed to increase output diversity during decoding (hence, in this case, the chemical diversity of candidate compounds). Conditional probabilities for SMILES tokens were derived by the Softmax function of the decoder.

The conditional transformer component was trained on a large number of target-compound triples (see below). The model was then applied to sample candidate (output) compounds for (*Protein sequence embedding, Potency embedding*) input instances.

### Tokenization

For model training, protein sequences, compounds, and potency values must be tokenized. Specifically, protein sequences were represented as standard uppercase residue symbols and tokenized using a single space. The vocabulary consisted of 21 tokens including the 20 natural amino acids plus “X” for rare amino acids. Compounds were encoded as canonical SMILES strings. Atoms were represented as single-character tokens (e.g., "C" or "N"), two-character tokens (e.g., "Cl" or "Br"), or tokens enclosed in brackets (e.g., "[nH]" or "[O-]"). Potency values were tokenized based on potency range binning [15, 16, 37]. Therefore, the globally observed potency range of [4.00, 12.52] pK_i_ units was divided into 852 bins with a constant width of 0.01. This granularity (resolution) captures the limits of experimental potency annotations. Each bin was encoded as a single token, and each potency value was assigned to the corresponding token. Additionally, two special tokens, i.e., "start" and "end," were defined to mark the beginning and end point of a sequence, respectively. This tokenization scheme was introduced previously for the successful generation of potent compounds [15].

### Model derivation and evaluation

The conditional transformer variant was trained using the Adam optimizer with a learning rate of 1e-5 and 1024 dimensions for the hidden states, thus precisely matching the settings of the ProtTrans PLM to prevent information loss through the connection. A batch size of 1 was chosen to place the longest protein sequence into GPU memory, and a gradient accumulation scheme was employed to achieve an effective batch size of 64. Training was carried out on a single NVIDIA Tesla A40 (48G) GPU. Throughout the training process, the cross-entropy loss between the ground truth and the output sequence was minimized. The model was trained for at least 50 epochs and at the end of each epoch, a checkpoint was saved. The final model was selected based on minimal cross-entropy loss. The training procedure included pre-training and fine-tuning.

The data set for model pre-training consisted of 212,004 target-compound pairs from 1565 activity classes. For each target-compound pair, triples were generated, as described above:

(*Protein sequence embedding, Potency embedding*) → (*Compound*).

For each pre-training and fine-tuning compound, its experimental potency value was embedded.

As a control, an unconditional transformer with the same architecture but without potency information was also derived from all compounds-target pairs:

(*Protein sequence embedding*) → (*Compound*).

**Fig. 2:**
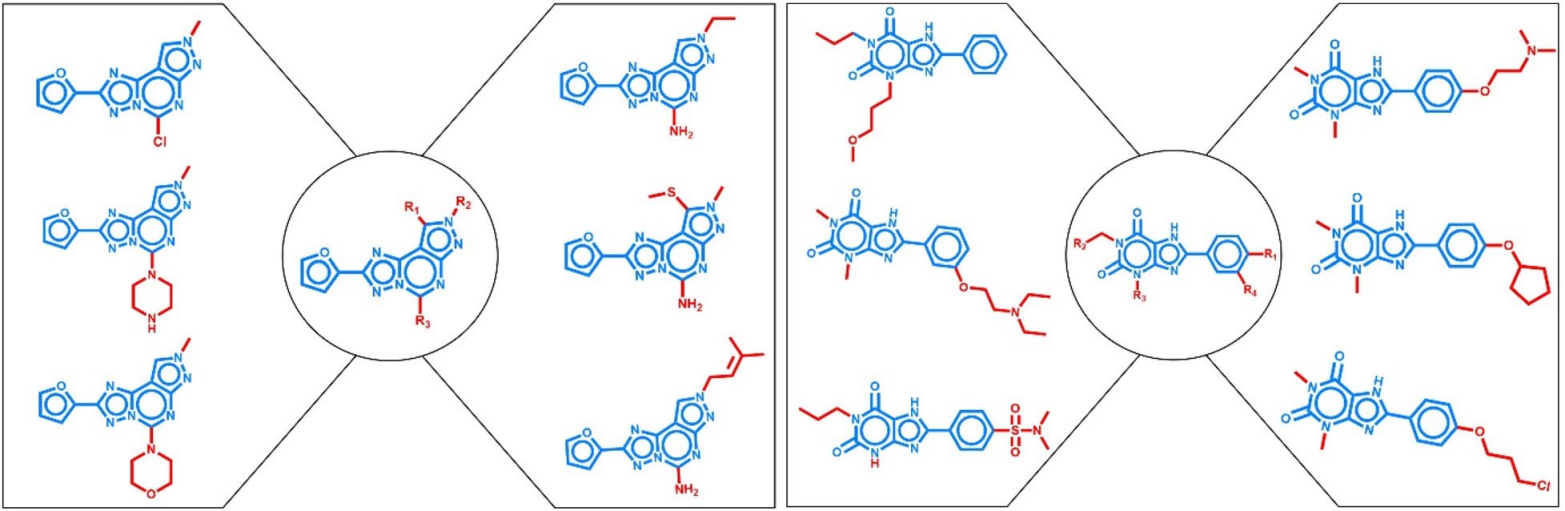
Exemplary analogue series. On the left and right, two distinct AS are shown consisting of six compounds each. In the center, the common core structure is displayed and all substitution sites are indicated. In the analogues, distinguishing substituents are colored red

For model fine-tuning and evaluation, each of the 10 activity classes in Table 1 was separately used. Importantly, model fine-tuning and testing were carried out on structurally distinct activity class subsets. Therefore, for each class, a systematic search for analogue series (AS) was conducted using the compound-core relationship (CCR) algorithm [38]. This method employs an extended modified matched molecular pair (MMP) fragmentation procedure [39] based on retrosynthetic rules [40] to systematically identify AS with single or multiple (up to five) substitution sites. The core structure of an AS was required to contain at least twice the number of non-hydrogen atoms of the combined substituents [38]. AS obtained for each activity class were then randomly divided into 50% fine-tuning and 50% test instances, ensuring no overlap in core structures between these sets. Consequently, the fine-tuning and test sets were structurally distinct. Figure 2 shows two exemplary AS.

For each test compound, a (*Protein sequence embedding, Potency embedding*) input instance was generated using its experimental potency value. Then, maximally 100 valid compounds (valid SMILES) were sampled, and these candidates were compared to all test compounds. The model's capacity to exactly reproduce known compounds was determined as the most stringent criterion for model validation. Additionally, for each activity class, 1-nearest neighbor (1-NN) similarity was calculated to compare the generated candidate compound structures with known test compounds. 1-NN similarity was quantified using the Tanimoto coefficient (Tc) [41], calculated based on 2048-bit Morgan fingerprints [42] with a bond radius of 3.

## Results and discussion

### Study concept

Our study had four primary objectives. (1) Conceptualize target-based compound generation as a machine translation task from a “protein language” to a “chemical language”. Therefore, protein representation learning was employed through the incorporation of a PLM. (2) Investigate if compound design across different activity classes could be facilitated on the basis of sequence-based protein representations (embeddings), without reliance on prior knowledge of ligand binding sites (for example, by defining characteristics sequence motifs of binding regions). (3) Evaluate the effects of potency value conditioning on generative compound design. (4) Assess model performance in a most rigorous manner. To address the first two objectives, which were central to our study, we designed a new dual-component conditional biochemical language model to process data of different modality. The model was challenged to learn mappings of protein embeddings conditioned on molecular potency values to active compounds. To address the third objective, we repeated the calculations using a corresponding unconditional model without context-dependent potency conditioning. To address the fourth objective, exact reproduction of known active compounds not encountered during training was set as the most stringent proof-of-concept criterion for the ability of the biochemical language model to correctly predict compounds with desired potency from protein sequence data. To this end, we ensured that fine-tuning and test sets for activity classes were structurally distinct by systematically identifying AS and partitioning them into non-overlapping subsets for fine-tuning and testing, respectively. There also was no compound overlap between activity classes.

### Reproducibility of known compounds

**Fig. 3:**
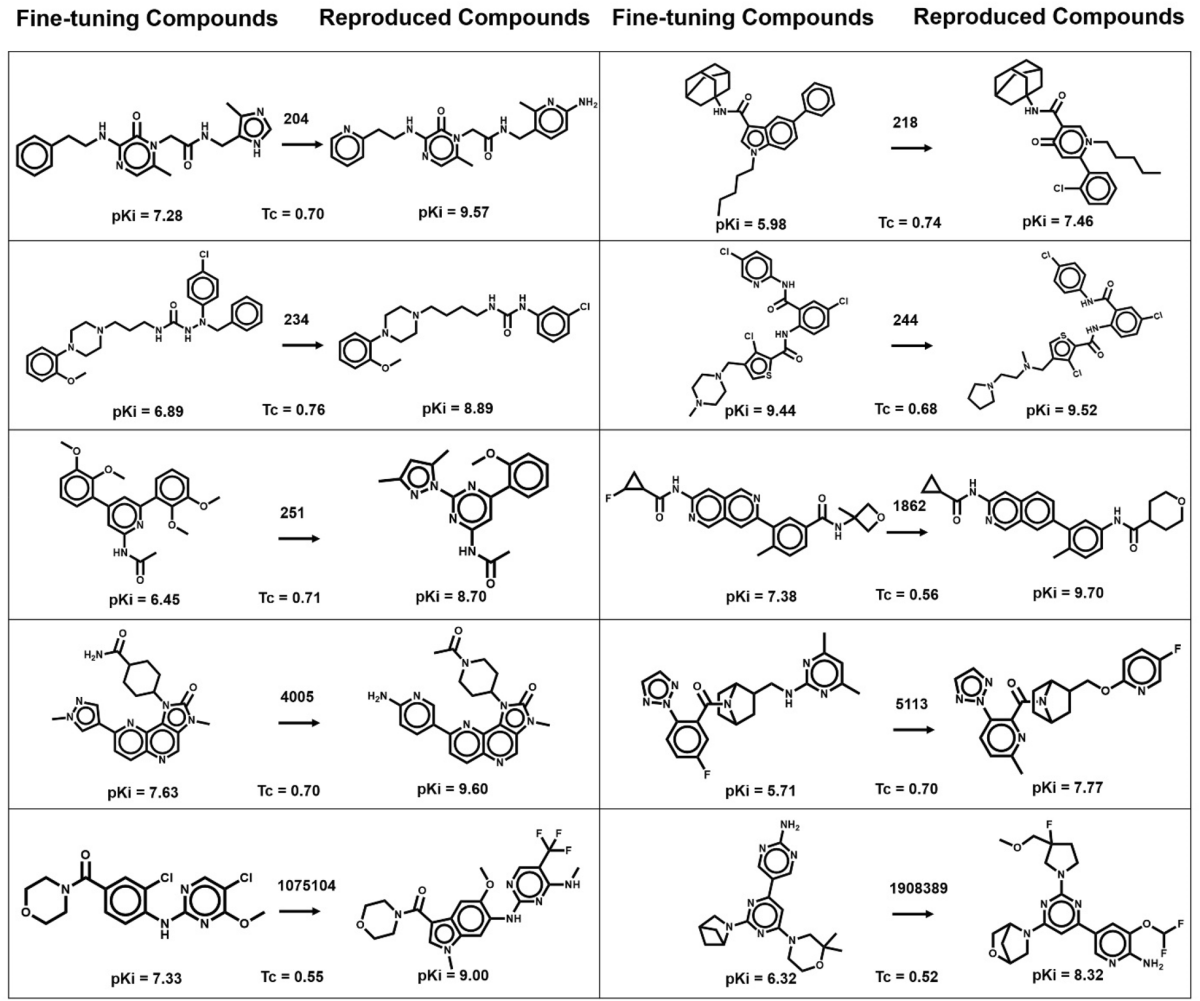
Exemplary predictions. For each activity class, exemplary test compounds are shown (right of the arrow) that were exactly reproduced using the conditional model together with the most similar fine-tuning compounds (left). For each test/fine-tuning compound pair, the Tanimoto similarity value is reported. ChEMBL IDs on arrows identify activity classes according to Table 1

The results of the systematic search for AS across 10 activity classes are presented in Table 2. The number of AS per activity class varied from 64 to 312 (“singleton” compounds not participating in any AS were disregarded). AS-based partitioning resulted in 74 to 619 compounds for fine-tuning and 318 to 1206 compounds for model evaluation, depending on the activity classes. In each case, AS were evenly divided (50/50%) and the subset with the smaller and larger total number of compounds was used for fine-tuning and testing, respectively. For each test instance, maximally 100 candidate compounds were sampled, canonicalized, and compared to compounds in the test set to identify exactly reproduced compounds. As reported in Table 2, both the conditional model and the unconditional model produced a substantial number of candidate compounds on the basis of target sequence embeddings. Specifically, depending on the activity class, the conditional model and unconditional model produced from 1789 to 7880 and from 769 to 4206 candidate compounds, respectively. As also reported in Table 2 (last two columns on the right), both the conditional and the unconditional model correctly reproduced multiple test compounds for each activity class; an encouraging finding. For the conditional model, the number of reproduced known compounds ranged from 10 to 115, with on average 43 per class, while the unconditional model generated between 3 and 57 known compounds, with on average 16 per class. Thus, the conditional model consistently reproduced ~ 2- to ~ 4-times more compounds per class than the unconditional model. By design, exact reproduction of test compounds ensured that these compounds had the desired potency value. Hence, these findings revealed a clear effect of compound potency conditioning on multimodal learning. Figure 3 shows exemplary predictions.

**Table 2:** Composition of fine-tuning and test sets and reproducibility of known active compounds

ChEMBL ID	Number of AS	Fine-tuning compounds	Test compounds	Sampled compounds		Reproduced compounds	
				Conditional	Unconditional	Conditional	Unconditional
204	130	134	320	2531	1181	16	4
218	250	285	833	2905	1730	75	29
234	213	499	1030	7880	4021	91	21
244	92	188	514	5163	1990	34	11
251	312	619	1206	7077	4206	115	57
1862	64	100	399	1789	894	21	7
4005	125	149	427	3592	2135	30	13
5113	155	288	798	3869	2021	25	10
1,075,104	114	74	323	1940	769	10	3
1,908,389	78	86	318	2324	1092	13	3

In Table 2, for each of 10 activity classes (ChEMBL target ID according to Table 1), the number of AS, number of compounds from AS for fine-tuning and testing, number of compounds produced by the conditional and unconditional model, and number of known test compounds exactly reproduced by the conditional and unconditional model are reported.

As a control, we also used the conditional model without fine-tuning to predict the test sets of three exemplary activity classes (204, 218, and 234). In these cases, the model sampled a total of 3082, 4328, and 8932 valid candidate compounds, respectively. However, no test compounds were reproduced in these calculations, as anticipated, thus confirming an essential role of class-specific fine-tuning.

### Potency value conditioning

**Fig. 4:**
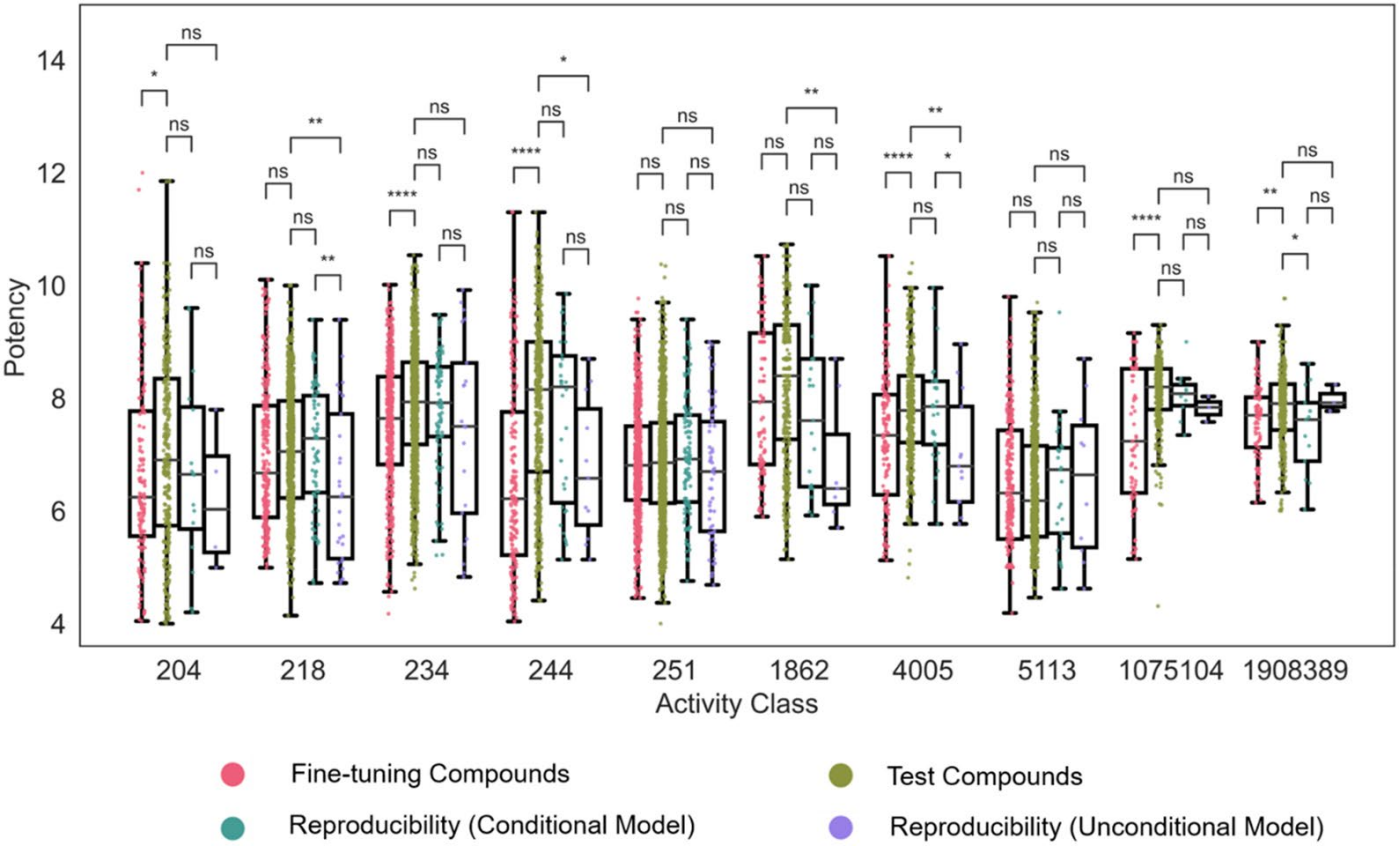
Potency value distributions of different compound subsets. For each activity class, boxplots compare logarithmic potency value distributions for all fine-tuning and test compounds and for test compounds correctly predicted by the conditional transformer and the unconditional model. To assess the statistical significance of differences between potency value distributions, independent-samples t-tests were conducted: 0.05 < p ≤ 1.00 (ns), 0.01 < p ≤ 0.05 (*), 0.001 < p ≤ 0.01 (**), 0.0001 < p ≤ 0.001 (***), p ≤ 0.0001 (****). Stars denote increasing levels of statistical significance and “ns” stands for “not significant”

In Fig. 3, exemplary pairs of reproduced compounds and their most similar fine-tuning compounds are shown for each activity class. In each pair, the reproduced compound is displayed on the right side of the arrow, and its most similar fine-tuning compound is on the left side. In addition, for each pair, the 1-NN similarity is reported, ranging from 0.52 to 0.76 depending on the activity classes. These examples illustrate the recurrent successful reproduction of test compounds from combined target sequence and compound potency embeddings. Moreover, the comparison of most similar fine-tuning and test compounds also indicated that test compounds correctly reproduced by the model had at least comparable, but often higher potency than the corresponding fine-tuning compounds. Notably, higher potency of predicted compared to fine-tuning compounds was not encoded as a conditional constraint. In Fig. 4, boxplots compare the potency value distributions of fine-tuning and test compounds from all activity classes with the potency value distributions of test compounds correctly predicted by the conditional transformer and the unconditional model.

The comparison showed that potency value distributions and the resulting median values of fine-tuning and test compounds differed depending on the activity class, as one would expect. In some instances, the median potency of test compounds was higher than of fine-tuning compounds and vice versa. However, for most activity classes, the potency distributions of test compounds correctly predicted by the conditional model closely matched the potency distributions of all test compounds, consistent with the desired effects of potency conditioning. By contrast, the unconditional model mostly reproduced smaller numbers of compounds with lower median potency than those correctly predicted by the conditional model, thus revealing a tendency to under-predict compound potency values in the absence of potency conditioning. Notably, the absence of statistical significance of potency differences between compounds reproduced with the conditional and unconditional model was mostly a consequence of the imbalanced sample sizes, including very small samples for the unconditional model (Table 2).

### Similarity analysis

**Fig. 5:**
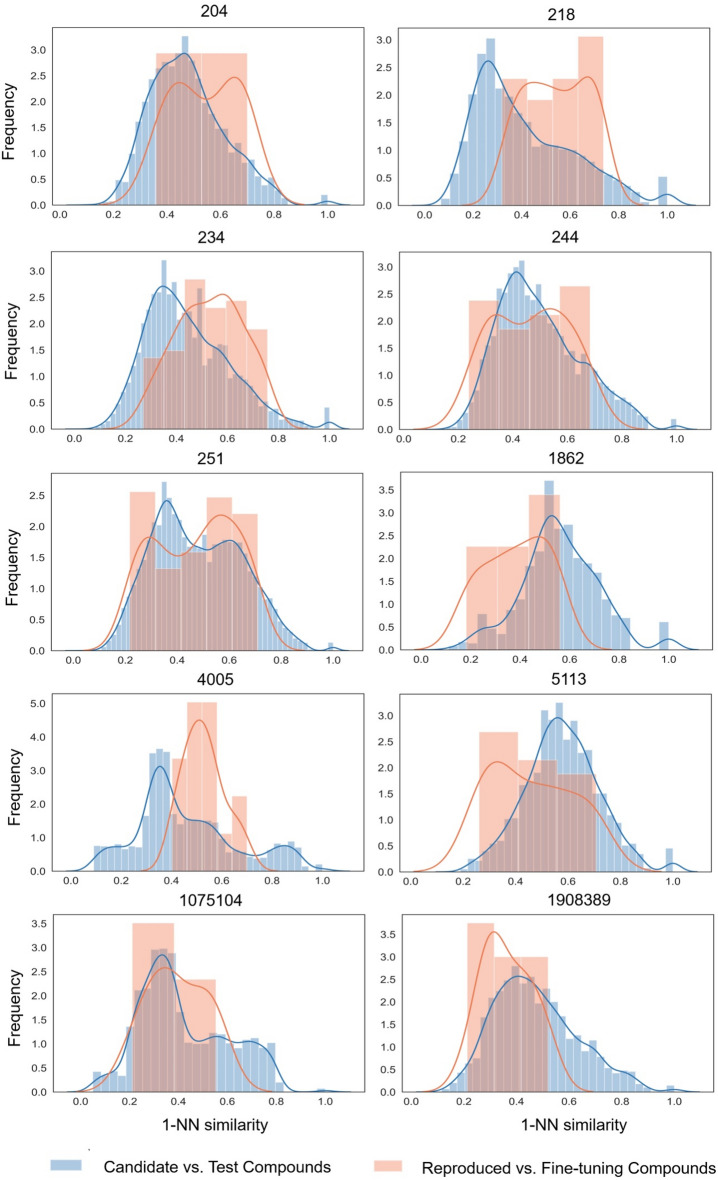
Distribution of 1-nearest neighbor similarities. For each activity class, blue and orange value distributions show 1-NN similarities of sampled candidate compounds vs. test compounds and correctly reproduced compounds vs. fine-tuning compounds, respectively

In addition to identifying and characterizing correctly reproduced test compounds, the 1-NN similarity of all sampled candidate compounds to test compounds was determined. Importantly, for rigorously establishing proof-of-concept of the approach, it was essential to confirm the ability of the biochemical language model to exactly reproduce known active compounds. However, for the practical relevance of the model and its design capacity, generalization potential should also be assessed. Ideally, a model with generalization ability should diversify candidate compounds (i.e., structurally abstract from fine-tuning and test compounds). Hence, the generation of candidate compounds with increasing structural diversity compared to known compounds also represented an important evaluation criterion. Therefore, we first systematically compared newly generated candidate compounds to test compounds. Figure 5 shows the distribution of 1-NN similarities of predicted candidate compounds compared to test compounds across the 10 activity classes. The predicted compounds consistently exhibited a variety of 1-NN similarities to test compounds, ranging from identical (or nearly identical) structures (100% 1-NN similarity) to distinct structures (~ 10% similarity). The most frequently observed 1-NN similarities ranged from ~ 30% to ~ 60%, depending on the activity class. These findings underscored the capability of the biochemical language model to not only reproduce known compounds but also generate structurally diverse candidate compounds.

Secondly, we also examined the distribution of 1-NN similarities for reproduced test compounds compared to fine-tuning compounds across the 10 activity classes. The reproduced compounds also exhibited a wide range of 1-NN similarities compared to fine-tuning compounds, from (~ 18%, ~ 56%) to (~ 40%, ~ 70%) across all activity classes. Here, the most frequently observed 1-NN similarities varied from ~ 25% to ~ 65%, depending on the activity class. Hence, these findings also confirmed the ability of the approach to abstract from fine-tuning compounds.

### Synthetic accessibility

**Fig. 6:**
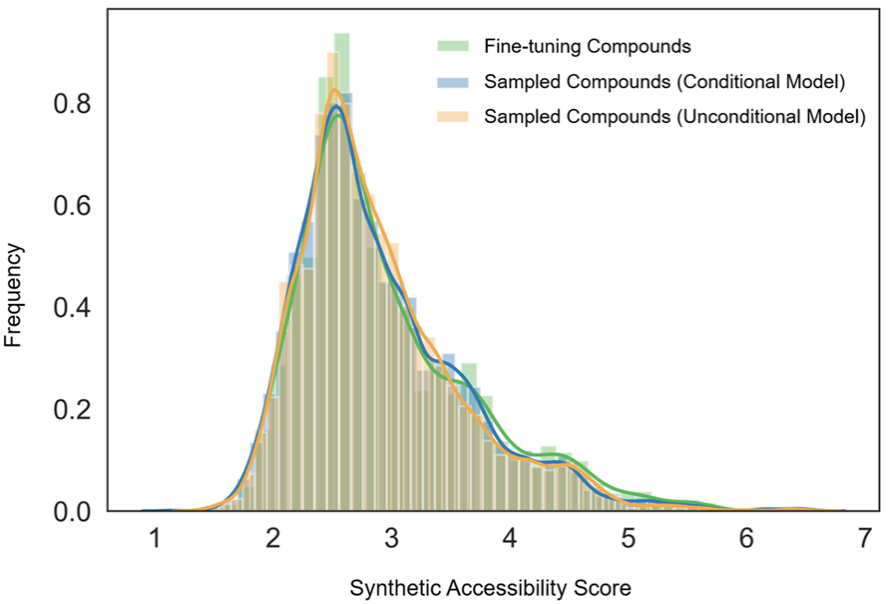
Synthetic accessibility. Compared are distributions of SA scores calculated for fine-tuning compounds and candidate compounds sampled with the conditional and unconditional model, respectively

While exact reproduction of known test compounds represents the ultimate criterion for establishing proof-of-concept for the design approach, newly generated candidate compounds also provide a resource for synthesis. Therefore, we have compared the synthetic accessibility (SA) of all sampled candidate compounds to the existing fine-tuning compounds using a well-established scoring scheme [43]. The results in Fig. 6 show that the SA score distributions for fine-tuning and candidate compounds sampled with both the conditional and unconditional model were nearly indistinguishable, thus indicating high SA for the newly generated candidate compounds.

## Conclusion

In this work, we have explored a new concept for predicting compounds with activity against given targets and desired potency from sequence embeddings with potency conditioning. For this purpose, a dual-component biochemical language model was designed for multimodal learning. The model included a pre-trained PLM (component 1) for protein representation learning and a conditional transformer (component 2) operating on the output of the PLM. The transformer was trained to learn mappings of target sequence embeddings conditioned on potency values to active compounds. Accordingly, the model input for generative design was heterogeneous, combining a sequence embedding with a molecular property constraint. The model was individually fine-tuned on 10 different target-based activity classes not included in model derivation. Model fine-tuning and evaluation were carried out on structurally distinct compound subsets generated by comprehensive AS identification and AS-based compound splitting. As the most rigorous proof-of-concept criterion for the approach, the ability of the biochemical language model to exactly reproduce known active compounds not encountered during training was determined. By design, exactly reproduced compounds had desired potency. The biochemical language model consistently reproduced varying numbers of known active compounds for all activity classes; an encouraging finding. Moreover, compared to an unconditional model used as a control, the conditional transformer consistently reproduced larger numbers of known compounds, thus revealing a clear positive effect of potency value conditioning on successful predictions. In addition, for most activity classes, the potency distribution of correctly reproduced compounds closely matched the potency distribution of all test compounds, consistent with reproducing compounds at different potency levels. Subsequent molecular similarity analysis showed that the biochemical language model was also capable of generating structurally diverse candidate compounds departing from both fine-tuning and test compounds; an indicator of model generalization potential.

Generative modeling compounds with desired potency from compound potency-conditioned target sequence embeddings was an unusual design task that might be expected to fail, for the scientific reasons discussed, and that could not possibly be addressed using standard ML approaches. Rather, for this challenging task, a language model was required to learn mappings of conditioned sequence data to active compounds, providing an example for a new potential opportunity provided by language models in compound design. Assessing whether or not such models might be predictive required a well-defined system set-up and rigorous evaluation criteria. The detected ability of the two-component biochemical language model to exactly reproduce compounds with pre-defined potency was not expected initially. Encouragingly, however, exact reproduction of test compounds was consistently observed across different activity classes, establishing proof-of-concept for such predictions.

Taken together, the results of our study suggest that compound design based on conditioned target sequence embeddings using language models merits further consideration. Currently, origins of correct compound reproduction remain model-internal and are non-transparent. Therefore, subsequent studies will be devised to explore the learning characteristics of the biochemical language model, rationalize correct predictions, and identify their input determinants. Furthermore, having established proof-of-principle at the methodological level, the approach will need to be prospectively assessed. For practical applications, it is straightforward, for example, to direct generative design towards highly potent compounds by setting corresponding potency thresholds. Furthermore, other context-dependent rules (such as different molecular property constraints) can be investigated in conjunction with target sequence embeddings. Moreover, the demonstrated ability of the biochemical language model to generate structurally diverse candidate compounds can also be explored in prospective applications by tesing new candidates. Therefore, given that the methodology is made freely available as a part of this study, there are ample opportunities for further research and applications.

## Data Availability

Calculations were carried out using publicly available programs and compound data. Python scripts generated for the study, the models, all pre-training and fine-tuning data, and newly generated compounds are available via the following link: https://uni-bonn.sciebo.de/s/Z9O2ZqKoA2cS7B1.

## Abbreviations

QSAR: Quantitative structure–activity relationship
ML: Machine learning
DL: Deep learning
CNN: Convolutional neural network
RNN: Recurrent neural network
GCN: Graph convolutional network
MPNN: Message passing neural network
SMILES: Simplified molecular input line entry system
PLM: Protein language model
AS: Analogue series
CCR: Compound-core relationship
1-NN: 1-Nearest neighbor
MMP: Matched molecular pair
Tc: Tanimoto coefficient
